# Learning-augmented optimization and control of long-haul mobility propulsion systems

**DOI:** 10.1016/j.isci.2026.115579

**Published:** 2026-04-02

**Authors:** Mubai Ding, Yisen Li, Chaoyi Chen, Nuo Lei, Hao Zhang

**Affiliations:** 1 School of Information Science and Engineering, Fudan University, Shanghai 200433, China; 2 Department of Electrical and Systems Engineering, University of Pennsylvania, Philadelphia, PA 19104, USA; 3 School of Vehicle and Mobility, Tsinghua University, Beijing 100084, China; 4 Department of Mechanical Engineering, Carnegie Mellon University, Pittsburgh, PA 15213, USA

**Keywords:** applied sciences, mechanical engineering, mechanical systems

## Abstract

Decarbonizing the long-haul transportation sector is a critical global challenge. This paper introduces a hierarchical, learning-augmented framework for co-designing an ammonia-hydrogen hybrid electric powertrain as a viable carbon-free propulsion solution. At the core of the proposed framework is the tight coupling between online control and offline design. In the online supervisory control layer, a deep reinforcement learning (DRL) agent is trained to make real-time decisions on power split and on-board hydrogen production. In the offline propulsion system optimization layer, a DRL-augmented adaptive non-dominated sorting genetic algorithm (RL-ANSGA) is employed to solve the multi-objective component sizing problem, with each candidate design evaluated in high-fidelity co-simulation under a fixed, pre-trained DRL energy management policy. Results demonstrate that the optimized ammonia-hydrogen vehicle outperforms conventional diesel and diesel-hybrid counterparts in energy efficiency and well-to-wheel carbon emissions.

## Highlights

• A hierarchical framework co-optimizes A-HEV design and real-time control
• DQN learns energy management strategies and on-board hydrogen production in real time
• RL-ANSGA adaptively improves multi-objective component sizing
• Optimized A-HEV beats diesel baselines in efficiency and WTW emissions

## Graphical abstract

**Figure:**
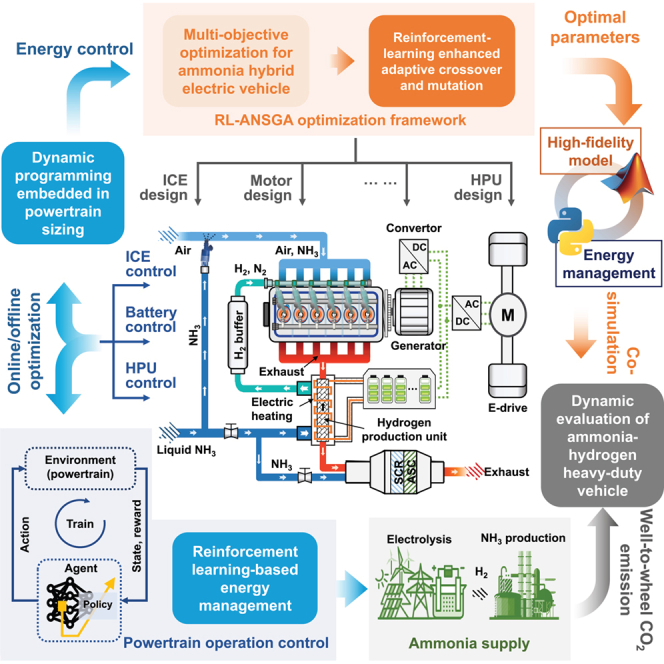

## Introduction

The imperative to achieve global energy conservation and carbon neutrality has intensified as anthropogenic carbon emissions reached a record of more than 35 Gt in 2023. This unprecedented level of emissions significantly erodes the remaining global carbon budget, creating a precarious scenario where the allowable emissions threshold could be breached within a span of up to 6 years, long before full de-carbonization is realized.^1^^,^^2^ According to the International Energy Agency (IEA), the transportation sector stands as a primary contributor, accounting for nearly a quarter of global carbon output, thus making it a critical focal point for emission control strategies.^3^^,^^4^ The internal combustion engine (ICE) remains the technological cornerstone of this sector—powering road transport, construction machinery, and maritime shipping—and is consequently a principal consumer of fossil fuels.^5^ Driven by the ambitious targets of international climate frameworks such as the Paris Agreement, the challenge of drastically reducing carbon emissions from ICEs has emerged as a shared and urgent mission for both academic research and industrial development.^6^

To confront this challenge, the scientific community has advanced two synergistic approaches: the adoption of clean, carbon-free fuels and the strategic electrification of power systems.^7^ Among potential zero-carbon fuels, hydrogen is often lauded for its exceptional gravimetric energy density and clean combustion characteristics. Specifically, Novella et al. explored the dilution potential for full-load operations, confirming hydrogen’s capability to power heavy-duty engines effectively.^8^ However, its path to widespread adoption is fraught with substantial logistical and technical obstacles, including difficulties in production, the lack of a mature transportation infrastructure, and the profound challenges of storing it on-board a vehicle safely and compactly.^9^^,^^10^ In stark contrast, ammonia presents itself as a highly pragmatic carbon-free fuel and hydrogen vector. Its distinct advantage lies in its ease of liquefaction, which allows for dense energy storage and transportation using existing infrastructure, thereby offering a more immediately viable pathway toward sustainable energy solutions, particularly with the rise of green ammonia production technologies.^11^^,^^12^^,^^13^ Recognizing ammonia’s potential to bridge the gap to a hydrogen economy, numerous countries have launched strategic initiatives to accelerate its development, underscoring its indispensable role in the future energy landscape.^14^

Nevertheless, the direct utilization of ammonia as a primary fuel in ICEs presents its own set of technical hurdles, most notably its high ignition energy and sluggish flame propagation speed.^15^ To overcome these combustion deficiencies, co-firing with a small amount of hydrogen has been identified as a highly effective enabling technology.^16^^,^^17^ This advanced combustion method enhances flame stability and dramatically accelerates the overall combustion process. A pivotal element of this concept is the ability to generate the required hydrogen on-board via a catalytic ammonia dissociation and separation unit (DSU), which cracks a portion of the stored ammonia into hydrogen and nitrogen.^18^^,^^19^^,^^20^ This integrated technology, especially when leveraging exhaust energy recovery, obviates the need for a separate hydrogen storage system and establishes an elegant pathway for a self-sufficient ammonia-hydrogen dual-fuel powertrain.^21^

While this powertrain architecture holds immense promise, its efficiency and viability are contingent on solving complex system-level challenges. The techno-economic feasibility of on-board hydrogen production via ammonia cracking requires careful consideration of catalyst costs and thermal management, necessitating advanced component optimization.^22^^,^^23^ Specifically, effective thermal design involves resolving conflicts between competing objectives, such as maximizing heat transfer while minimizing pumping power.^24^^,^^25^ Furthermore, embedding this ammonia-hydrogen engine within a hybrid electric drive system—a promising approach for heavy-duty applications^26^^,^^27^^,^^28^^,^^29^—promises to eliminate operational carbon emissions entirely. However, the application of ammonia fuel, though widely studied in various contexts including combined heat and power systems,^30^^,^^31^^,^^32^ has not been met with sufficient research into the holistic optimization of the entire power system. The performance of such a complex system depends critically on the synergistic matching of its key components, including the DSU, generator, electric motors, and power batteries.^33^ Beyond component matching, effective system control is vital. Wang et al. applied dynamic programming, yet it is computationally demanding and requires future knowledge.^34^ Meanwhile, Saiteja and Ashok noted that simple rule-based strategies often yield suboptimal economy under dynamic conditions.^35^

Optimization techniques have been widely studied to enhance the performance of complex engineering systems, ranging from thermal management structures with complex geometries to heavy-duty propulsion systems.^36^^,^^37^^,^^38^ However, a fundamental research gap persists in this domain: the lack of a systemic approach to the co-design of the powertrain hardware and its associated control strategy. The optimal sizing of physical components is deeply intertwined with the real-time energy management policy that governs power flow between the engine, battery, and DSU.^39^^,^^40^ Treating these as independent design problems—optimizing hardware first, then designing a controller, or vice versa—inevitably leads to a globally suboptimal system, failing to exploit the full energy-saving capacity.^41^ To address the limitations of traditional approaches, data-driven methods have gained prominence. Li et al. and He et al. systematically reviewed deep reinforcement learning (DRL) for hybrid powertrains. They demonstrated that DRL agents can effectively handle high-dimensional non-linear dynamics, offering a promising alternative to traditional optimization methods for complex energy management tasks.^42^^,^^43^ While advanced, learning-based control strategies from various research groups have demonstrated significant potential for optimizing energy efficiency in real-time,^44^^,^^45^^,^^46^ their full benefit can only be realized when the underlying hardware is designed in concert with them.^47^ Research is urgently needed to address this co-optimization challenge for carbon-free propulsion systems, integrating real-time control within the system design loop.

This paper directly confronts these challenges by conducting a systematic study to unlock the full potential of ammonia-hydrogen propulsion systems. We make several key contributions to the field. First, leveraging detailed experimental data on the combustion and emission characteristics of an active hydrogen jet ignition ammonia engine, we construct and validate a high-fidelity ammonia-hydrogen hybrid electric powertrain (A-HEV) model, including the on-board ammonia cracking unit (DSU).^48^ Second, to overcome the limitations of traditional, decoupled optimization, we develop and implement an integrated design framework that combines an advanced genetic algorithm with reinforcement learning to holistically co-optimize the system’s hardware parameters and control policy. Finally, we provide a comprehensive analysis of the resulting optimized system, including its dynamic energy flow, waste heat recovery, and well-to-wheel (W2W) carbon emissions, providing a crucial perspective in the context of broader energy system assessments.^49^^,^^50^ Through this work, we demonstrate the significant potential of our intelligent design framework and methodology to advance energy conservation and accelerate carbon reduction in the long-haul heavy-duty transport sector.

## Results

### A hierarchical, learning-augmented framework for powertrain co-design

At the core of our investigation is an innovative, hierarchical framework engineered for the integrated design and optimization of the ammonia-hydrogen hybrid powertrain. This architecture is founded upon the principle of hardware-software co-design, a methodology that addresses the intrinsic coupling between the physical powertrain components and their operational control logic. To manage this complexity, we leverage artificial intelligence (AI) across two distinct but interconnected tiers: an offline tier for multi-objective hardware parameter optimization and an online tier for real-time, intelligent operational control.

**Figure 1:**
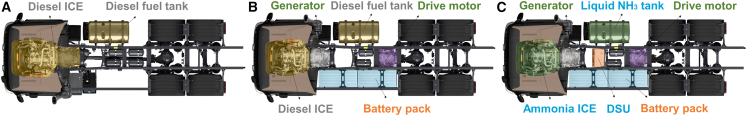
Powertrain architectures Comparison of (A) a conventional diesel vehicle (D-ICEV), (B) a hybrid electric diesel vehicle (D-HEV), and (C) the proposed hybrid electric vehicle fueled with ammonia-hydrogen (A-HEV). Key components are highlighted, including the diesel fuel tank, battery pack, generator, drive motor, liquid NH_3_ tank, and the ammonia DSU.

To establish a rigorous basis for comparison, we first define the candidate powertrain architectures, as depicted in Figure 1. The proposed ammonia-hydrogen hybrid electric vehicle (A-HEV) is systematically evaluated against two critical benchmarks: the conventional diesel ICE vehicle (D-ICEV), representing the industry incumbent, and the diesel hybrid electric vehicle (D-HEV), representing the current state-of-the-art in hybrid technology for heavy-duty applications. This comparative setup provides a clear and comprehensive context for quantifying the performance gains and environmental benefits of our proposed solution.

**Figure 2:**
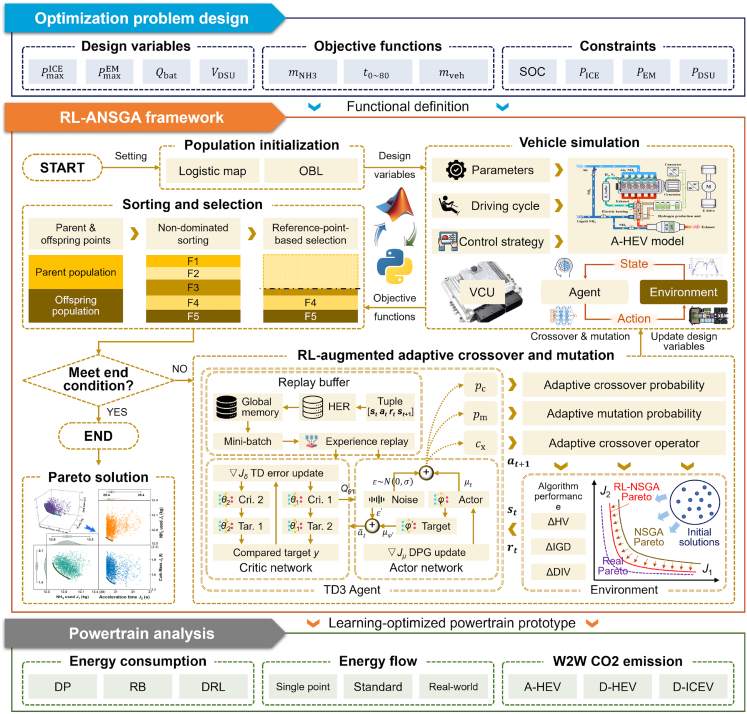
Reinforcement learning-augmented heuristic optimization for powertrain sizing The overall structure of the RL-ANSGA framework. The optimization problem is defined by design variables, objective functions, and constraints. The framework uses an adaptive NSGA-III algorithm where the genetic operators are controlled by a DRL agent. The agent’s policy is trained based on the evolutionary algorithm’s performance metrics (ΔHV, ΔIGD, and ΔDIV). Each candidate design is evaluated through a co-simulation loop involving a high-fidelity A-HEV model, which is controlled by a pre-trained energy management agent. The final output is a Pareto-optimal set of solutions.

The upper tier of our AI-driven framework is dedicated to solving the complex, multi-objective design problem of component sizing (Figure 2). For this task, we introduce an optimization algorithm, termed the RL-ANSGA. While traditional evolutionary algorithms like NSGA-II and neural network-based surrogates have proven effective in optimizing various complex thermal and energy systems,^51^^,^^52^^,^^53^ the vast, high-dimensional, and conflicting nature of the heavy-duty powertrain design space makes it challenging for these methods to navigate efficiently. Our RL-ANSGA enhances the conventional NSGA-III process by embedding a DRL agent that acts as a meta-controller. As a standard co-optimization baseline, we also adopt a conventional NSGA-III procedure that jointly optimizes the component sizing vector and the tunable parameters of a rule-based energy management system (EMS), following the formulation and workflow reported in our prior work.^48^ This baseline represents a widely used GA-based co-optimization workflow for high-fidelity powertrain design. In RL-ANSGA, the meta-controller dynamically modulates the genetic operators—specifically, the crossover probability, mutation probability, and the selection of the operator type—during the evolutionary search. The RL agent is trained to progressively improve the optimization process itself, using feedback from key multi-objective performance indicators like the change in hypervolume (ΔHV) and inverted generational distance (ΔIGD). This meta-optimization layer enables the algorithm to intelligently balance the trade-off between exploration of the search space and exploitation of promising regions, leading to a more efficient and robust convergence toward the true Pareto-optimal set of hardware parameters.

**Figure 3:**
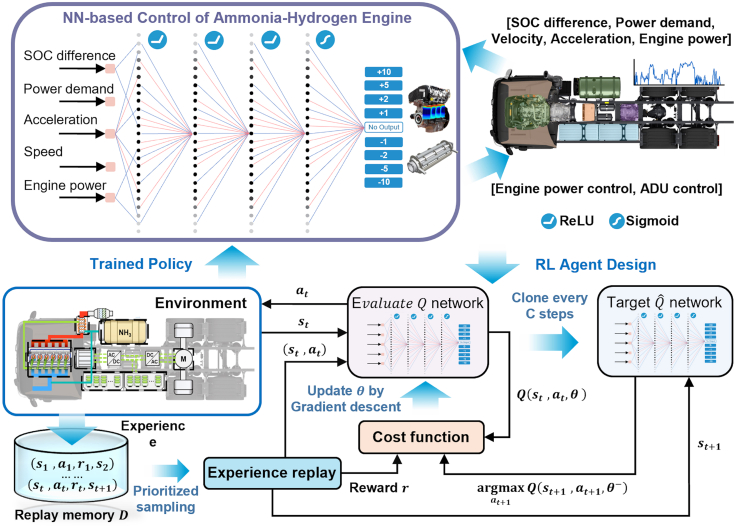
Reinforcement learning-based energy management strategy The training process for the DQN-based EMS agent. The agent interacts with the powertrain environment (the high-fidelity simulation model). Transitions (state, action, reward, next state) are stored in a replay memory. Mini-batches are sampled from this memory to update the Q-network via gradient descent, minimizing a cost function. A separate target Q-network is used to stabilize training. The final trained policy is a neural network that maps vehicle states to optimal control actions for the ammonia-hydrogen engine and DSU.

The lower tier of the framework focuses on the synthesis of an intelligent online control policy, as detailed in Figure 3. Once a specific set of hardware parameters is defined (either as a candidate solution during the offline optimization or as the final selected design), a sophisticated RL-based EMS is deployed. This EMS must navigate the complex, real-time trade-offs between minimizing fuel consumption, maintaining battery charge sustainability, and managing the thermal state of the ammonia DSU. To address this, we employ a deep Q-network (DQN), a value-based DRL algorithm, to train a control agent. This agent learns an optimal policy through extensive interaction with a high-fidelity simulation of the powertrain environment. The policy maps observable vehicle states (such as velocity, acceleration, and battery state-of-charge) to optimal control actions, governing both the power split between the engine and battery and the power allocated to the DSU for on-board hydrogen production. Crucially, this highly proficient, pre-trained DQN policy serves as the intelligent “brain” for evaluating the fitness of each candidate hardware design within the outer RL-ANSGA optimization loop. This nesting of the online controller within the offline design process effectively closes the loop on hardware-software co-design, ensuring that the optimized physical system is perfectly synergistic with its intelligent control strategy.

**Figure 4:**
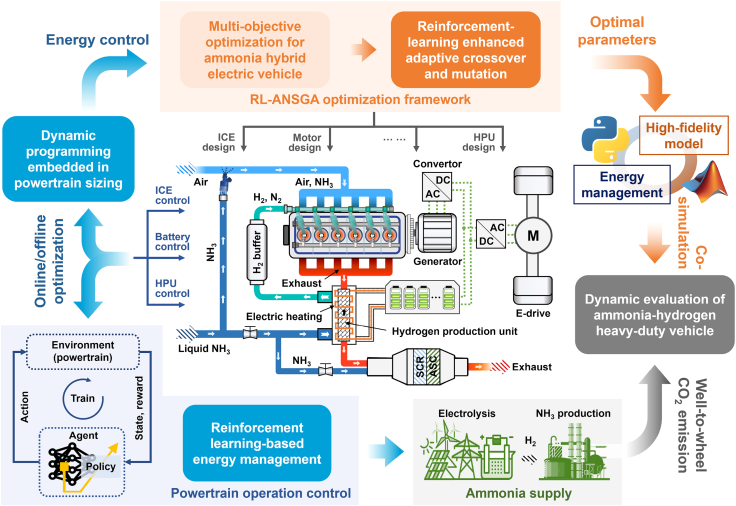
Schematic of the learning-augmented optimization and control framework This integrated framework demonstrates the hierarchical approach. The outer loop, the RL-ANSGA optimization framework, performs multi-objective hardware sizing to find optimal parameters for the ICE, motor, battery, and HPU. The inner loop relies on a pre-trained reinforcement learning-based EMS for the dynamic evaluation of each candidate design. The final optimized system is then assessed for energy consumption and W2W carbon emissions, considering the entire chain from ammonia supply to vehicle operation.

The complete, integrated co-design architecture is schematically illustrated in Figure 4. The process commences with the outer-loop, multi-objective optimization of powertrain component sizes, which is intelligently guided by the RL-ANSGA algorithm. Each candidate design generated by the algorithm is then passed to the inner loop, where it is virtually instantiated in the simulation environment and operated by the pre-trained RL-based EMS over a range of representative driving conditions. The performance metrics from this dynamic evaluation are fed back to the RL-ANSGA as fitness scores. This iterative process continues until the solution set converges, yielding a Pareto front of hardware parameters that are co-optimized with a near-optimal control policy. The final selected powertrain is then subjected to a rigorous and comprehensive post hoc analysis of its energy consumption dynamics, internal energy flows, and complete well-to-wheel (W2W) carbon emissions, providing a holistic and definitive assessment of its operational performance and sustainability profile.

### System dynamics and optimization performance

**Figure 5:**
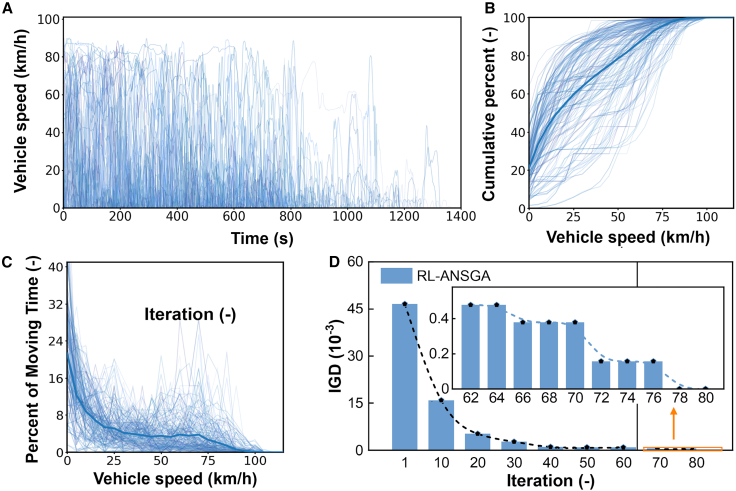
Driving cycles and optimization convergence (A) Speed profiles from a collection of real-world heavy-duty vehicle driving cycles. (B) Cumulative speed distribution of the collected cycles. (C) Percentage of moving time spent at various speeds. (D) Convergence of the IGD metric during the RL-ANSGA optimization process. The rapid decrease in IGD demonstrates the algorithm’s efficiency in approaching the true Pareto front. The inset shows the stabilization of IGD in the later generations.

To ensure our simulations and the resulting control policies are robust and reflective of real-world conditions, our framework was grounded in a comprehensive dataset of driving cycles meticulously collected from in-use heavy-duty vehicles (Figures 5A–5C). These empirical data, which encompass a wide spectrum of operational speeds, accelerations, and road grades, serve as the foundational environment for both training the EMS agent and evaluating the performance of candidate powertrain designs. For policy learning and validation, the collected driving cycles are randomly partitioned into a 70% training subset and a 30% held-out testing subset with no overlap. The testing subset is reserved for all subsequent performance evaluation to assess generalization on unseen driving conditions. The use of such diverse, real-world data is critical for developing a controller that can generalize effectively beyond standardized test cycles.

The efficacy of our proposed RL-ANSGA optimization process is evidenced by its strong convergence behavior, as illustrated by the evolution of the inverted generational distance (IGD) metric in Figure 5D. The IGD value, which quantifies the distance between the algorithm’s current set of solutions and the true (or best-known) Pareto front, exhibits a rapid and monotonic decrease. The initial sharp decline corresponds to the exploration phase, where the meta-learning agent guides the search toward promising regions, while the later, more gradual stabilization of the metric indicates a fine-tuning or exploitation phase. This trajectory confirms that the algorithm efficiently and effectively identifies a high-quality approximation of the optimal design trade-offs.

**Figure 6:**
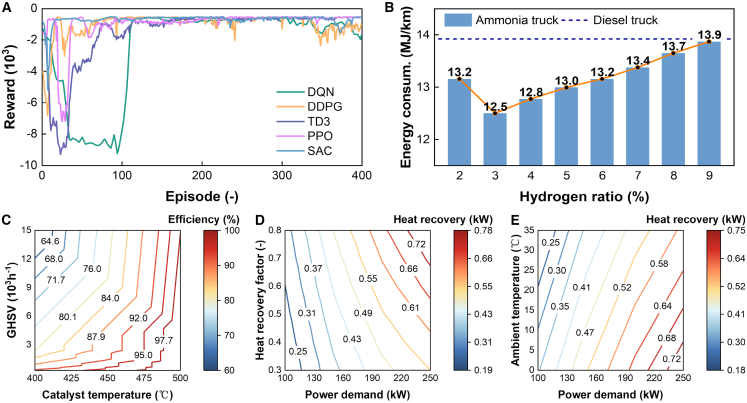
EMS training and system performance analysis (A) Cumulative reward during the training of the EMS agent for different DRL algorithms (DQN, DDPG, TD3, PPO, and SAC). (B) Energy consumption of the A-HEV (bar chart) compared to a diesel baseline (dashed line) across different hydrogen ratios, with the DRL-based EMS. The orange line shows the percentage improvement. (C) DSU conversion efficiency as a function of catalyst temperature and GHSV. (D) Waste heat recovery potential as a function of the waste heat recovery coefficient and vehicle power demand at an ambient temperature of 25°C. (E) Waste heat recovery potential as a function of ambient temperature and vehicle power demand with a fixed recovery coefficient of 0.5.

The training of the DQN-based EMS agent also proved highly effective. Figure 6A presents the learning curves, obtained on the training subset, plotting the cumulative reward over training episodes for several state-of-the-art DRL algorithms. While multiple algorithms show learning capability, the DQN and soft actor-critic (SAC) agents demonstrate particularly stable and high-performance convergence, validating the applicability of DRL to this complex control problem. The final A-HEV, optimized using this framework and equipped with the trained DRL-based EMS, demonstrates a marked advantage in energy efficiency over a conventional diesel counterpart. The quantitative performance results and comparisons reported in Figures 6B–6E are evaluated exclusively on the held-out testing subset, which contains driving cycles unseen during training. As shown in Figure 6B, there exists a distinct optimal hydrogen mass ratio of approximately 3%. Below this ratio, combustion efficiency may degrade, while above it, the significant energy penalty associated with ammonia cracking outweighs the combustion benefits, leading to higher overall energy consumption.

A deeper analysis of the DSU’s thermal characteristics reveals further avenues for system enhancement. The DSU’s conversion efficiency is a complex function of catalyst temperature and gas hourly space velocity (GHSV), as mapped in Figure 6C. Furthermore, the potential for recovering waste heat from the engine’s exhaust to power the endothermic decomposition reaction is substantial, varying with ambient temperature and vehicle power demand (Figures 6D and 6E). These characterizations are not merely academic; they provide critical data for both the online EMS, which must manage DSU operation, and the offline RL-ANSGA, which determines the optimal size of the DSU and heat exchange components. Beyond the hydrogen mass ratio, Figures 6C–6E also indicate sensitivity to DSU/thermal parameters: DSU conversion efficiency varies with catalyst temperature and GHSV, while waste-heat recovery potential changes with recovery coefficient, ambient temperature, and power demand. These trends suggest robustness depends on managing DSU thermal inertia and leveraging buffer-assisted coordination in the EMS.

**Figure 7:**
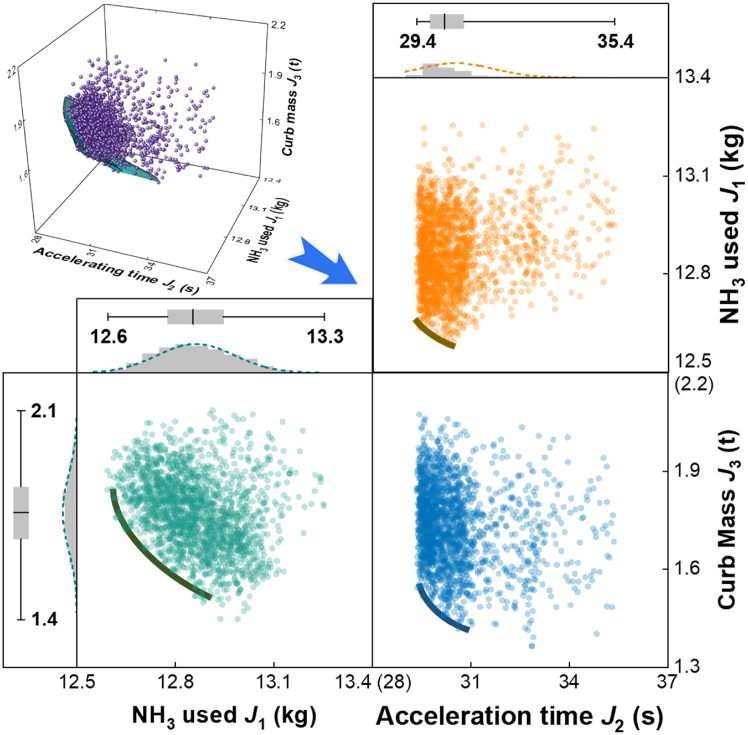
Pareto front of the multi-objective optimization The results of the RL-ANSGA optimization showing the trade-offs between the three objectives. The 3D plot shows the overall Pareto surface. The 2D projections illustrate the specific relationships between pairs of objectives: vehicle mass vs. acceleration time (top right), ammonia consumption vs. vehicle mass (bottom left), and ammonia consumption vs. acceleration time (bottom right). Histograms show the distribution of solutions for each objective.

The final, synthesized output of the RL-ANSGA optimization is the Pareto front shown in Figure 7. This multi-dimensional surface visualizes the fundamental trade-offs between the three conflicting design objectives: minimizing ammonia consumption (fuel economy), minimizing acceleration time (performance), and minimizing vehicle curb mass (cost and payload capacity). Such a representation is an invaluable tool for engineers, as it transforms the abstract optimization problem into a tangible design space. It allows stakeholders to select a specific hardware configuration that consciously balances these competing attributes according to the priorities of a given application, whether it be long-haul freight (prioritizing fuel economy) or vocational tasks (prioritizing performance).

### Analysis of energy flow and carbon emissions

**Figure 8:**
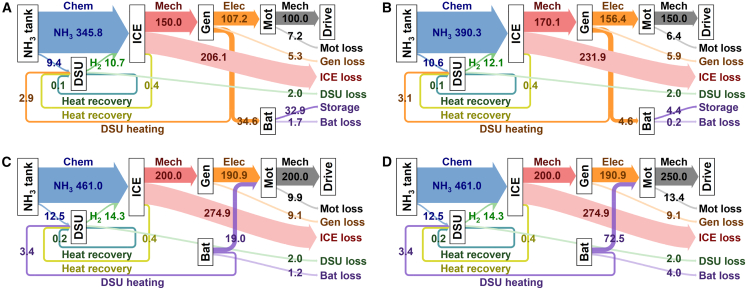
Energy flow distribution under various steady-state power demands Sankey diagrams showing the energy flow (in kW) at demands of (A) 100 kW, (B) 150 kW, (C) 200 kW, and (D) 250 kW. The diagrams trace energy from the chemical source (fuel tank) through the ICE, DSU, generator (Gen), battery (Bat), and motor (Mot) to the final drive, quantifying losses at each stage.

To gain deeper insight into the operational behavior of the optimized system, a detailed energy flow analysis was conducted under both steady-state and dynamic conditions. Under steady-state operation, with power demands ranging from 100 to 250 kW, the Sankey diagrams in Figure 8 provide a transparent illustration of the energy distribution pathways. These diagrams reveal the intelligence of the EMS in action: at lower power demands (e.g., 100 kW), surplus energy from the ICE is efficiently routed to charge the battery, effectively shifting the engine’s operating point to a region of higher efficiency. Conversely, at peak power demands (e.g., 250 kW), the EMS seamlessly coordinates the discharge of the battery to supplement the engine’s output, meeting the demand without oversizing the ICE. This load-leveling capability is a hallmark of a well-managed hybrid system.

**Figure 9:**
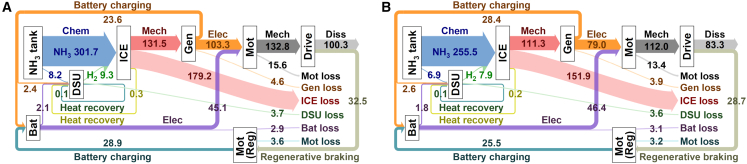
Energy distribution during standard driving cycles Sankey diagrams showing the total energy flow (in MJ) for the (A) CWTVC and (B) CHTC cycles. The diagrams account for energy consumed from the tank, converted by the engine, stored/released by the battery, and recovered through regenerative braking.

Under transient conditions representative of real-world driving, the energy flow analysis over the China world transient vehicle cycle (CWTVC) and China heavy-duty commercial vehicle test cycle (CHTC) (Figure 9) quantifies the total energy budget. These diagrams meticulously account for every joule of energy, from the chemical energy in the ammonia tank to its conversion, storage, losses in various components, recovery through regenerative braking, and final delivery to the wheels for propulsion. This holistic accounting provides a complete picture of the system’s integrated efficiency across realistic and challenging driving scenarios.

**Figure 10:**
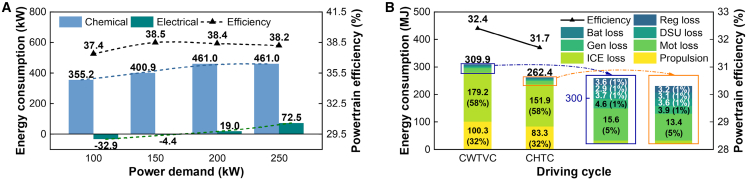
System efficiency and energy distribution analysis (A) System energy consumption and efficiency across different power points. Bars represent chemical energy input and battery energy change, while the dashed line shows the resulting system efficiency. (B) Overall energy distribution breakdown for the CWTVC and CHTC cycles, showing the percentage of initial fuel energy converted to propulsion (drive), lost through the ICE, or lost in other components.

The overall system efficiency, plotted in Figure 10A, reaches a peak of approximately 38.5% under optimal steady-state conditions. The comprehensive energy breakdown for the dynamic driving cycles, shown in Figure 10B, is particularly revealing. While approximately 32% of the initial fuel energy is successfully converted into useful propulsion, a substantial 58% is lost as thermal energy in the exhaust gas. This figure, while representing a loss, simultaneously highlights a significant opportunity for future advancements in waste heat recovery technologies, which could further enhance the system’s overall efficiency.

**Figure 11:**
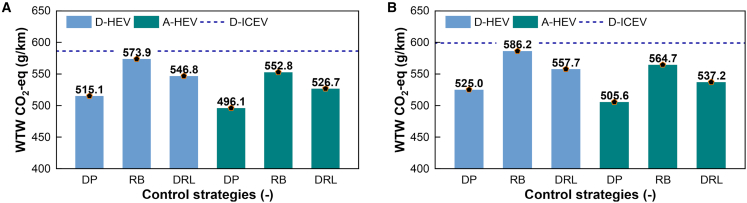
Well-to-wheel (WTW) CO_2_ equivalent emissions Comparison of WTW CO_2_-eq (g/km) for different vehicle powertrains (D-ICEV, D-HEV, and A-HEV) with various EMS (dynamic programming, DP; rule-based, RB; deep reinforcement learning, DRL) under (A) the CWTVC and (B) CHTC driving cycles.

Finally, the ultimate measure of the system’s environmental performance is its W2W carbon equivalent emissions, presented in Figure 11. The results unequivocally demonstrate that the optimized A-HEV achieves consistently lower W2W emissions than both the D-ICEV and D-HEV benchmarks across all tested driving cycles and control strategies. Most notably, the A-HEV equipped with our DRL-based EMS achieves near-optimal performance. This helps avoid conflating controller limitations with optimizer performance. This result is a powerful validation of our hierarchical, learning-based approach. It demonstrates the dual and compounding benefits of combining a cleaner powertrain architecture with a deeply intelligent control strategy to minimize environmental impact and forge a viable path toward decarbonized long-haul transportation.

## Discussion

This study culminates in a successful demonstration of the technical viability and substantial decarbonization potential of an ammonia-hydrogen hybrid powertrain, a feat made possible by a hierarchical AI-driven design framework. The quintessential innovation of this work lies in the symbiotic integration of online intelligent control with offline hardware optimization. This approach directly addresses a persistent and critical gap in the co-design of complex energy systems, effectively solving the “chicken-and-egg” dilemma where the optimal hardware configuration depends on its control strategy, and the optimal control strategy is a function of the hardware. By first architecting a near-optimal, real-time energy management strategy using DRL, we establish a high-performance “software” baseline. This intelligent controller is then ingeniously employed as a dynamic fitness evaluator within a sophisticated, RL-augmented multi-objective evolutionary algorithm to discover the optimal “hardware” configuration. A common baseline is to size components using a genetic algorithm and evaluate candidates with a rule-based EMS. This can bias the sizing outcome toward a suboptimal controller and confound attribution. We therefore use a fixed pre-trained learning-based EMS as a consistent evaluator during the sizing optimization. This holistic methodology ensures that the final system is not merely a collection of well-chosen components, but a deeply synergistic entity where hardware and software are mutually optimized.

The tangible benefits of this hardware-software co-design philosophy are clearly manifested in the results. The final optimized A-HEV exhibits not only superior fuel economy but also achieves the lowest well-to-wheel CO_2_ equivalent emissions when benchmarked against both conventional diesel and state-of-the-art diesel-hybrid systems. The comprehensive energy flow and performance analyses afford profound insights into the system’s intricate operational dynamics. They underscore the pivotal role of the intelligent EMS in masterfully orchestrating the complex interplay of energy flows—deftly load-leveling the engine, managing the battery’s state-of-charge, and satisfying the thermal demands of the onboard hydrogen production unit—to sustain peak efficiency across a wide array of demanding, real-world operating conditions. From deployment standpoint, DSU thermal inertia slows response versus electrical power split. Our supervisory EMS regulates DSU output/heating and tracks hydrogen buffer to bridge traction transients. Hardware delays and rate limits can be modeled as lags/constraints and enforced via runtime safety filtering, motivating HIL/vehicle validation. Concurrently, the RL-ANSGA framework demonstrated exceptional efficacy, successfully navigating the vast, non-convex, and multi-modal design space to deliver a well-defined and practical Pareto front. This outcome provides engineers with clear, actionable intelligence, enabling them to make informed decisions by transparently balancing the inherent trade-offs between vehicle performance, operational efficiency, and overall system mass.

The methodological paradigm presented herein is characterized by its broad applicability and is not confined to the specific powertrain investigated. It offers a powerful, scalable, and adaptable framework for the systematic design and assessment of other next-generation energy systems where the interplay between physical configuration and intelligent control is paramount. Potential applications range from hybrid fuel-cell vehicles and advanced maritime propulsion to the management of microgrids and industrial energy systems. This work, therefore, represents a significant conceptual and practical step forward, providing a robust blueprint for applying advanced AI techniques to de-risk and accelerate the global transition toward a sustainable and fully decarbonized transportation future.

### Limitations of the study

While this study presents a comprehensive and robust framework, a candid acknowledgment of its limitations is essential for contextualizing the results and guiding future research. The primary limitations and corresponding avenues for extension are as follows:

First, the fidelity of the component models, though grounded in empirical data, does not explicitly capture the long-term degradation dynamics. Effects such as battery capacity fade due to cycling, gradual deactivation of the catalyst in the DSU, and mechanical wear on the ICE are not incorporated. Future investigations should endeavor to integrate physics-based or data-driven aging models into the simulation environment. This would enable optimization not just for initial performance but for lifetime efficiency and reliability, which is a critical consideration for commercial viability.

Second, the DRL-based control agent was trained and evaluated on a specific, albeit extensive, dataset of real-world driving cycles. While this approach promotes realism, the agent’s performance may vary when deployed in operational domains with statistical characteristics significantly different from the training data, such as unique terrains or payload conditions. Future work could explore advanced machine learning techniques, such as transfer learning or continual learning, to enhance the controller’s adaptability, allowing it to fine-tune its policy and maintain near-optimal performance when confronted with environments.

Finally, the W2W analysis, while crucial, is predicated on the carbon intensity of current, predominantly fossil-fuel-based ammonia production pathways. The global ammonia industry is on the cusp of a major transformation toward “green” (renewable-powered electrolysis) and “blue” (natural gas with carbon capture) production methods. The framework presented is inherently adaptable; future research should leverage this flexibility by updating the life cycle assessment with evolving emission factors from these greener pathways. This would provide a more dynamic and forward-looking evaluation of the powertrain’s long-term environmental credentials as the global energy infrastructure evolves. At present, most ammonia supply is still produced from unabated fossil pathways, and while low-emission hydrogen and ammonia projects are expanding, a large fraction of announced capacity remains at early development stages.^54^^,^^55^ Key barriers to accelerating adoption include access to low-cost renewable electricity and electrolyser scale-up, as well as the establishment of certified low-carbon supply chains and safe distribution and refueling standards for end-use sectors.^56^

## Resource availability

### Lead contact

Requests for further information and resources should be directed to and will be fulfilled by the lead contact, Hao Zhang (haoz4@andrew.cmu.edu).

### Materials availability

This study did not generate new materials.

### Data and code availability

• All data reported in this paper will be shared by the lead contact upon reasonable request.
• The original code used for simulation and optimization is available upon request.
• Any additional information required to reanalyze the data reported in this paper is available from the lead contact upon request.

## Acknowledgments

The authors acknowledge funding and support from 10.13039/501100001809National Natural Science Foundation of China (grant no. 52302410, grant no. U24B20124). We also thank the members of our research group for their insightful discussions and support.

## Author contributions

Conceptualization, N.L. and H.Z.; methodology, M.D., Y.L., and C.C.; software, M.D. and Y.L.; validation, M.D. and Y.L.; formal analysis, M.D. and Y.L.; investigation, M.D. and Y.L.; writing – original draft, M.D. and Y.L.; writing – review and editing, N.L. and H.Z.; funding acquisition, C.C. and H.Z.; supervision, C.C. and H.Z.

## Declaration of interests

The authors declare no competing interests.

## STAR★Methods

### Key resources table

**Table:** 

REAGENT or RESOURCE	SOURCE	IDENTIFIER
**Software and algorithms**		

Python	Python Software Foundation	https://www.python.org
MATLAB	MathWorks	https://www.mathworks.com

### Experimental model and study participant details

There are no experimental model or study participants to include in this study.

### Method details

#### Bi-level optimization for Co-Design

The central theoretical contribution of this work is the formalization of the ammonia-hydrogen powertrain design challenge as a mathematically rigorous bi-level optimization problem. This structure is uniquely suited to capture the intrinsic coupling between the offline decisions of hardware sizing and the online dynamics of software control, providing a principled, systems-engineering approach to their co-design. This problem can be conceptualized as a Stackelberg game, wherein an upper-level “leader” (the system designer) selects an optimal hardware configuration while anticipating the optimal response of a lower-level “follower” (the real-time supervisory control system).

The upper-level problem is a multi-objective optimization task that seeks to identify the optimal hardware design vector, **x**. This vector comprises key physical parameters, including the maximum power ratings of the ICE and electric motor $\left(P_{\text{ICE}}^{\max},P_{\text{EM}}^{\max}\right)$, the total capacity of the battery pack (*Q*_bat_), and the catalyst volume of the DSU (*V*_*DSU*_). The objective is to simultaneously minimize a vector of conflicting performance metrics, **F**(**x**). The core challenge resides in the fact that the performance of any given hardware design, **x**, is critically dependent on the intelligence of its control policy, denoted by *π*. Consequently, the upper-level objective function is defined as the performance achieved under the ∗optimal∗ control policy for that specific hardware, *π*∗(**x**). The upper-level problem is therefore formulated as:

(Equation 1) $$\min_{x\in X}\;F\left(x,\pi^{*}\left(x\right)\right)$$

subject to a set of physical, operational, and economic constraints that define the feasible design space, X. The objective vector **F** includes total ammonia consumption, 0-80 km/h acceleration time, and overall vehicle mass.

The lower-level problem is to determine this optimal control policy, *π*∗(**x**), for a fixed hardware design, **x**, provided by the upper level. This constitutes a stochastic optimal control problem, with the goal of maximizing the expected cumulative reward, *J*, over a representative distribution of driving cycles, D:

(Equation 2) $$\pi^{*}\left(x\right)=\arg\;\underset{\pi }{\max}\;J\left(\pi ;x\right)=E_{C∼D}\left[\overset{T}{\underset{t=0}{\sum }}\gamma^{t}r_{t}\left(s_{t},a_{t}\right)|\pi ,x\right]$$

where *s*_*t*_ is the system state at time *t*, *a*_*t*_ is the control action taken, *r*_*t*_ is the instantaneous reward received, and *γ* is the temporal discount factor.

Our solution methodology is a hierarchical, agent-driven framework that mirrors this bi-level structure. We first develop a generalizable method to solve the lower-level problem for any given hardware vector **x** using DRL. This process yields a single, robust DQN-based EMS policy $\pi_{\mathrm{DQN}}^{*}\left(x\right)$ trained offline to generalize across the feasible hardware-design space. For notational convenience, we denote the performance evaluation of this fixed policy under a given design **x** as $\pi_{\mathrm{DQN}}^{*}\left(x\right)$, without retraining the policy for each candidate design. Subsequently, we solve the upper-level problem using a RL-augmented meta-heuristic algorithm. In this stage, the computationally expensive evaluation of the objective function for any candidate design **x** is performed through a high-fidelity co-simulation controlled by the fixed pre-trained DQN policy $\pi_{\mathrm{DQN}}^{*}\left(x\right)$. In the outer-loop evaluation, the controller is used for inference only and is not retrained for each candidate design.

#### High-fidelity powertrain simulation model

The co-simulation environment, which serves as the virtual testbed for both optimization levels, is constructed upon a detailed, physics-based model of the powertrain. Each subsystem is modeled with a level of fidelity that balances accuracy with computational tractability. Empirical efficiency maps, derived from extensive dynamometer testing, are used to model the engine and drivetrain, ensuring that the model accurately reflects the real-world performance and loss characteristics of the specific hardware. The DSU’s thermodynamics and chemical kinetics are captured using an Arrhenius-based reaction rate embedded in a dynamic energy balance, enabling the model to represent its coupled thermal–chemical behavior. For the battery, a first-order Thévenin equivalent-circuit model is employed to capture the essential electrical dynamics, including state-of-charge-dependent open-circuit voltage. This electrical model is coupled with a thermal model to account for internal heat generation and its impact on temperature-dependent internal resistance. Finally, a standard vehicle longitudinal dynamics model calculates the required power at the wheels based on vehicle speed, acceleration, road grade, and aerodynamic drag. This high-fidelity composite model serves as the state transition function, $P\left(s_{t+1}|s_{t},a_{t};x\right)$, for the Markov decision processes (MDPs) at both levels of the optimization hierarchy.

#### Lower-level DRL energy management

The lower-level task of deriving an optimal real-time energy management strategy is formally cast as a MDP and solved using a DQN agent.

#### MDP formulation for energy management

The MDP is formally defined by the tuple (S,A,P,R,γ), where each component is tailored to the energy management problem:

• **State Space** (S)**:** The state, $s_{t}\in S\;\subset \;R^{4}$, provides an example of representation of the system’s instantaneous condition required for optimal decision-making. It includes vehicle velocity (*v*_*t*_), acceleration (*a*_*t*_), battery state-of-charge (SOC_*t*_), and the mass of hydrogen in the buffer storage (*m*_*H*2,*buff*_): *s*_*t*_ = [*v*_*t*_, *a*_*t*_, SOC_*t*_, *m*_*H*2,*buff*_]. All state variables are normalized to the interval [0, 1] to facilitate stable training of the neural network approximator.
• **Action Space** (A)**:** To leverage the power of value-based DRL methods like DQN, the inherently continuous control space is discretized into a finite set of actions, *a*_*t*_ ∈ {1, ..., *N*_*a*_}. Each discrete action corresponds to a specific combination of commands for the engine’s output power, DSU output power (controlling hydrogen production rate) and the DSU’s heating power. This discretization strikes a critical balance between maintaining fine control fidelity and ensuring the stability and convergence of the DQN algorithm.
• **State Transition Dynamics** (P)**:** The transition dynamics, $s_{t+1}∼P\left(s_{t+1}|s_{t},a_{t}\right)$, are not modeled explicitly. Instead, they are implicitly defined by the complex, non-linear interactions within the high-fidelity powertrain simulation model described previously.
• **Reward Function** (R)**:** The reward function is meticulously engineered to encapsulate the control objectives. It guides the agent towards minimizing fuel consumption while simultaneously maintaining charge sustainability and adhering to physical operational constraints. It can be formulated as a weighted, multi-component function:

(Equation 3) $$r_{t}=-w_{1}\;\cdot \;{\dot{m}}_{{\text{NH}}_{3}}\left(t\right)-w_{2}\;\cdot \;{\left(\text{SOC}\left(t\right)-{\text{SOC}}_{\text{target}}\right)}^{2}-w_{3}\;\cdot \;1_{\text{DSU}\;\text{violation}}$$

where the first term penalizes instantaneous ammonia consumption, the quadratic second term imposes a smooth penalty for deviations from the target SOC, and the indicator function **1**(⋅) applies a large discrete penalty for violating the hydrogen buffer storage limits of the DSU, ensuring operational safety.

#### DQN-based policy synthesis

We employ the DQN algorithm, a cornerstone of modern DRL, to approximate the optimal action-value function, *Q*∗(*s*, *a*). The theoretical foundation for this approach is the Bellman optimality equation, which recursively defines the value of an action in a given state. DQN uses a deep neural network, *Q*(*s*, *a*; *θ*), as a powerful function approximator for *Q*∗(*s*, *a*). This network is trained by minimizing the temporal difference (TD) error using stochastic gradient descent. The loss function at each iteration *i* is:

(Equation 4) $$L\left(\theta_{i}\right)=E_{\left(s,a,r,s^{\prime }\right)∼U\left(D\right)}\left[{\left(r+\gamma \underset{a^{\prime }}{\max}\;Q\left(s^{\prime },a^{\prime };\theta_{i}^{-}\right)-Q\left(s,a;\theta_{i}\right)\right)}^{2}\right]$$

where *D* represents the experience replay buffer, and $\theta_{i}^{-}$ are the weights of a separate, periodically updated target network. The use of a replay buffer to store and sample past transitions and a target network to provide a stable TD target are two critical innovations that decorrelate data and stabilize the learning process. The resulting trained policy, $\pi_{\mathrm{DQN}}^{*}$, provides a robust, computationally efficient, and near-optimal EMS suitable for any given hardware configuration.

#### Upper-level search with learned policy

The upper-level hardware sizing problem is a computationally expensive, black-box, multi-objective optimization challenge. The objective function, **F**(**x**, *π*∗(**x**)), lacks an analytical form, and each evaluation is costly, requiring a full vehicle simulation. These characteristics render traditional gradient-based optimization methods infeasible. We therefore address this challenge using a meta-heuristic search, which we enhance with a meta-learning layer to intelligently guide the search process.

#### RL-ANSGA model

We propose the RL-ANSGA framework, which innovatively reframes the process of tuning the genetic algorithm’s operators as a sequential decision-making problem that can be learned.

• **Theoretical Rationale:** The performance of an evolutionary algorithm such as NSGA-III is widely recognized to be highly sensitive to the parameterization of its genetic operators. The No Free Lunch theorems for optimization further imply that no single parameter configuration can be uniformly optimal across problem classes, or even throughout different phases of a single optimization run. We therefore hypothesize that a desirable operator policy should be non-stationary and conditioned on the current state of the evolving population. Accordingly, we formulate the learning of this adaptive policy as a Markov decision process (MDP).
• Meta-Control MDP Formulation:
  – **State (*s***_***g***_**):** At each generation *g*, a meta-agent observes a state vector, *s*_*g*_, that provides a comprehensive, quantitative characterization of the population’s current status. The state vector is defined as *s*_*g*_ = [gen_norm_, HV_*g*_, IGD_*g*_, *σ*_crowd_, feas_ratio_], where gen_norm_ is the normalized generation number indicating search progress; HV_*g*_ is the hypervolume indicator, capturing both the convergence and diversity of the Pareto front; IGD_*g*_ is the indicator primarily reflecting convergence quality; *σ*_crowd_ is the standard deviation of the crowding distances, measuring solution diversity; and feas_ratio_ is the ratio of feasible solutions, indicating how effectively the search is navigating constraint boundaries.
  – **Action (*a***_***g***_**):** The meta-agent’s action is a continuous vector, *a*_*g*_ = [*p*_*c*_, *p*_*m*_, *o*_*sel*_], that defines the crossover probability (*p*_*c*_), mutation probability (*p*_*m*_), and the choice of specific genetic operators for the subsequent generation.
  – **Reward (*r***_***g***_**):** The reward is defined as the measured improvement in the quality of the Pareto front from one generation to the next: *r*_*g*_ = *α* ⋅ΔHV_*g*_ − *β* ⋅ΔIGD_*g*_*.* This reward structure directly incentivizes the agent to make decisions that accelerate convergence to a high-quality set of solutions.
• **Adaptive Operator Control via TD3:** We adopt the twin delayed deep deterministic policy gradient (TD3) algorithm as the meta-controller. TD3 is an actor–critic method designed for continuous action spaces, which makes it well suited for adjusting the continuous genetic-operator parameters *p*_*c*_ and *p*_*m*_*.* Relative to DDPG, TD3 incorporates several key refinements—clipped double Q-learning, delayed policy updates, and target policy smoothing—that jointly reduce Q-value overestimation and improve training stability. These properties make TD3 a robust choice for learning an adaptive control policy for sensitive genetic-operator settings.

#### Co-simulation for objective evaluation

The execution of the framework unfolds as an iterative dialogue between the outer-loop designer (RL-ANSGA) and the inner-loop controller (DQN), embodying the bi-level structure.

1. The outer loop consists of one generation of the RL-ANSGA, which maintains and evolves a population of candidate hardware designs, {**x**_*i*_}.
2. For each candidate design, **x**_*i*_, that requires evaluation, the inner loop is invoked. This involves programmatically parameterizing the high-fidelity Simulink model with the vector **x**_*i*_, loading the pre-trained DQN controller $\pi_{\mathrm{DQN}}^{*}$, and executing a detailed simulation over a representative driving cycle.
3. The key performance metrics from the simulation (e.g., total ammonia consumption, acceleration time) are then returned to the outer loop, serving as the objective function values, $F\left(x_{i},\pi_{\mathrm{DQN}}^{*}\right)$.
4. The RL-ANSGA uses these fitness values to perform selection and breeding. Simultaneously, the TD3 meta-agent observes the change in Pareto front quality, receives its reward, and updates its own policy to select a potentially more effective set of genetic operators for the next generation.

This tightly integrated process iterates until the Pareto front converges, yielding a final set of hardware designs that are certifiably co-optimized with a highly proficient, intelligent control strategy.

### Quantification and statistical analysis

This study primarily reports quantitative results from high-fidelity simulation, reinforcement learning training, and multi-objective optimization. Quantitative comparisons were performed using fuel consumption, component sizing results, Pareto-front quality indicators, and well-to-wheel CO2 emissions under the specified driving cycles and operating conditions.

### Additional resources

There are no additional resources to include in this study.
